# A deep position-encoding model for predicting olfactory perception from molecular structures and electrostatics

**DOI:** 10.1038/s41540-024-00401-0

**Published:** 2024-07-17

**Authors:** Mengji Zhang, Yusuke Hiki, Akira Funahashi, Tetsuya J. Kobayashi

**Affiliations:** 1 School of Biomedical Engineering, Shanghai Jiao Tong University, Shanghai, China; 2 Institute of Industrial Science, The University of Tokyo, Tokyo, Japan; 3 Department of Biosciences and Informatics, Keio University, Yokohama, Japan

**Keywords:** Computational science, Computational biology and bioinformatics, Computer science

## Abstract

Predicting olfactory perceptions from odorant molecules is challenging due to the complex and potentially discontinuous nature of the perceptual space for smells. In this study, we introduce a deep learning model, Mol-PECO (**Mol**ecular Representation by **P**ositional **E**ncoding of **Co**ulomb Matrix), designed to predict olfactory perceptions based on molecular structures and electrostatics. Mol-PECO learns the efficient embedding of molecules by utilizing the Coulomb matrix, which encodes atomic coordinates and charges, as an alternative of the adjacency matrix and its Laplacian eigenfunctions as positional encoding of atoms. With a comprehensive dataset of odor molecules and descriptors, Mol-PECO outperforms traditional machine learning methods using molecular fingerprints and graph neural networks based on adjacency matrices. The learned embeddings by Mol-PECO effectively capture the odor space, enabling global clustering of descriptors and local retrieval of similar odorants. This work contributes to a deeper understanding of the olfactory sense and its mechanisms.

## Introduction

Olfaction is one of the essential senses, where the sense of smell is triggered by the binding of odorant molecules to olfactory receptors and is shaped by the subsequent neural processing of the received information in the brain^1,2^. Unlike vision and hearing, however, the prediction of olfactory perception for odorant molecules remains challenging. On the one hand, some molecules with different functional groups share identical smells^3^. On the other hand, other molecules with similar structures can produce totally different perceptions^4^. The structures and perceptions of the odorant molecules are nonlinearly and discontinuously related^5^. Unveiling the quantitative structure–odor relationship (QSOR) is indispensable for understanding the coding principle of olfactory information^5^ and also essential for predicting and designing smells and flavors for various applications such as food technologies^6^.

Machine learning (ML) is a promising approach to untangle such a complicated relationship. However, the prediction of olfactory perception from molecular structures is strongly dependent on molecular representation^7^. Molecular descriptors, which encode chemical substructures into fixed-length vectors, are the major and classical molecular representation, yet demonstrate limited performances in QSOR due to the inefficient feature extraction by hand-crafted rules^8,9^. To learn a good representation of molecules from data, graph convolutional networks (GCNs) have been widely applied in molecular modeling^10^, e.g., quantum chemistry^11–13^, biophysics^14,15^, and biological side effects^12,15^. The conventional GCN models each molecule by the adjacency matrix, which encodes the chemical bonds as a graph, and performs the information aggregation among the neighbors prescribed by the adjacency matrix^12,16^. While GCN has been reported to outperform conventional ML with molecular fingerprints in standard tasks^16^, there still exist technical drawbacks, which potentially limit the applicability of GCN and the adjacency matrix^17–19^ to learning QSOR. First, the adjacency matrix cannot encode the atomic and global 3D information of molecules^7^, even though such atomic and 3D information is the major determinant of binding affinities between odorant molecules and olfactory receptors^20^. Second, graphs do not have a canonical coordinate representation, which contrasts with sequences and images for which one-, two-, and three-dimensional lattice coordinates are canonical. As a result, the GCN employs permutation invariant operations, e.g., message passing or neighboring aggregation, which then limit its expressive power to discriminate molecules with different structures^17^ and also induces oversmoothing^18^ and oversquashing^19^. All of these factors may hinder the network in efficiently learning the QSOR of odorant molecules with a variety of structures.

**Fig. 1:**
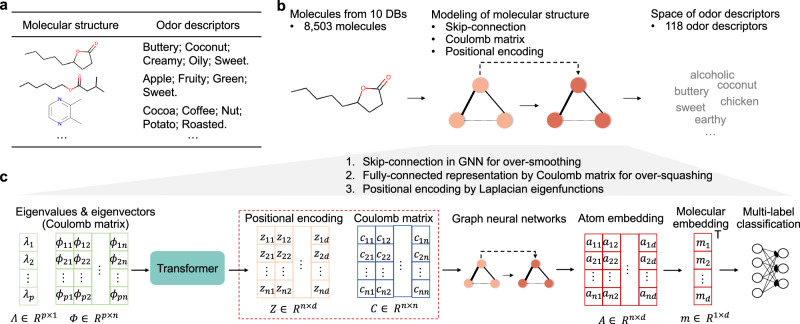
Overview of Mol-PECO. **a** Typical molecular structures and the corresponding odor descriptors are shown as examples. **b** The main workflow of modeling quantitative structure–odor relationship (QSOR). **c** The detailed model architecture of Mol-PECO and its three features: (1) skip-connection in graph neural networks to alleviate oversmoothing, (2) fully connected molecular representation by Coulomb matrix to suppress oversquashing, and (3) positional encoding by Laplacian eigenfunctions.

In this work, we develop a deep learning model (Mol-PECO) for QSOR, which aims at the multilabel classification of olfactory perception from molecular structures (Fig. 1). To address the problems in conventional ML and GCN approaches, Mol-PECO combines the Coulomb matrix (CM) and Spectral Attention Network (SAN). CM is a simple global representation of a molecule using Coulombic forces between atoms in the molecule calculated with the nuclear charges and the corresponding 3D coordinates^21^. Therefore, CM could encode more detailed structural information than the adjacency matrix which only represents the chemical bonds between atoms. SAN is an architecture of graph attention network (GAT)^17^, which uses the Laplacian spectrum of a molecular graph for a learned positional encoding (LPE). The eigenfunctions of the graph Laplacian hierarchically describe the global and local structures in a graph and thereby provide a way to characterize graphs and to define the positional information of nodes (atoms)^22^. SAN can also be categorized as an attempt to extend expressive power in graph-based architectures endowing graphs with coordinates or positional information by using their spectral information^17,22,23^. CM can be naturally combined with SAT by regarding CM as a weighted adjacency matrix.

Based on the learned representation, Mol-PECO directly predicts 118 odor descriptors of perception for each odorant molecule. Mol-PECO achieves the area under the receiver operating characteristic curve (AUROC) of 0.813 and area under the precision-recall curve (AUPRC) of 0.181, whereas the conventional MLs of molecular fingerprints fail to balance AUROC and AUPRC; the ML method (cfps-KNN) with the highest AUROC of 0.761 has a low AUPRC of 0.057 and one (mordreds-RF) with the highest AUPRC of 0.144 shows a low AUROC of 0.723. Thus, Mol-PECO may boost the prediction of QSOR for applications and also contribute to the understanding of the principle underlying olfactory information processing.

## Results

In this section, we first describe the dataset used in this work. Then, we introduce Coulomb-GCN, which updates GCN by replacing the adjacency matrix with CM. After the effectiveness of Coulomb-GCN is verified, we have Mol-PECO by further replacing the random embedding of atoms in Coulomb-GCN with positional encoding, where the spectral information of the CM is employed to have a structure-aware embedding.

### Dataset: a comprehensive human olfactory perception dataset

In this work, we use the data in which each molecular structure is paired with multiple odor descriptors (Fig. 1a). The dataset in our work is compiled from ten expert-labeled sources: Arctander’s dataset (*n* = 3102)^24^, AromaDb (*n* = 1194)^25^, FlavorDb (*n* = 525)^26^, FlavorNet (*n* = 718)^27^, Goodscents (*n* = 6158)^28^, Fragrance Ingredient Glossary (*n* = 1135)^29^, Leffingwell’s dataset (*n* = 3523)^30^, Sharma’s dataset (*n* = 4006)^31^, OlfactionBase (*n* = 5105)^32^, and Sigma’s Fragrance and Flavor Catalog (*n* = 871)^33^. These datasets were retrieved from the archive of https://github.com/pyrfume/pyrfume-data. The data cleaning procedure includes (1) merging overlapped molecules, (2) filtering conflict descriptors, and (3) filtering the rare descriptors assigned to less than 30 molecules. After data cleaning, we obtain a comprehensive dataset of 8503 molecules and 118 odor descriptors.

**Fig. 2:**
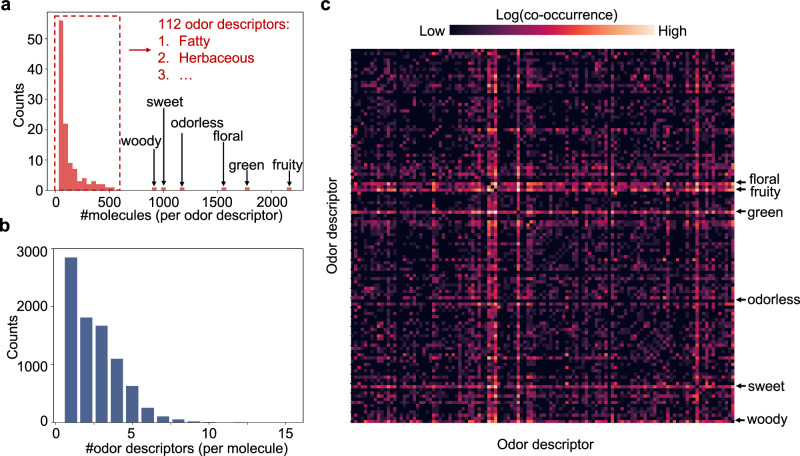
The comprehensive human olfactory dataset. **a** Distribution of molecules across odor descriptors. **b** Distribution of descriptors across molecules. **c** Co-occurrence matrix of 118 odor descriptors. The heatmap is demonstrated with logarithm transformation, and the descriptors are ordered alphabetically.

This comprehensive human olfactory perception dataset is multilabeled, with every molecule labeled with one or several odor descriptors. For the number of molecules associated with each odor descriptor, the distribution is imbalanced: each of the 112 odor descriptors is assigned to ≤800 molecules whereas the other 6 descriptors are linked to >800 molecules (Fig. 2a). For the number of descriptors associated with each molecule, the distribution is also skewed, with 8054 molecules possessing ≤5 odor descriptors and 449 molecules possessing >5 odor descriptors (Fig. 2b). For co-occurrence, the descriptors “fruity”, “green”, “sweet”, “floral”, and “woody” co-occur with almost all the descriptors, while odorless molecules co-occur with no other molecules (Fig. 2c). The data split is built by second-order iterative stratification^34^, which aims at splitting multilabel dataset and preserves the label ratios in each split with an iterative sampling design. The whole dataset is split into train/validation/test datasets of 6802/864/837 pairs, respectively.

### Fully connected graph by Coulomb matrix is superior to sparse graph by the adjacency matrix

**Fig. 3:**
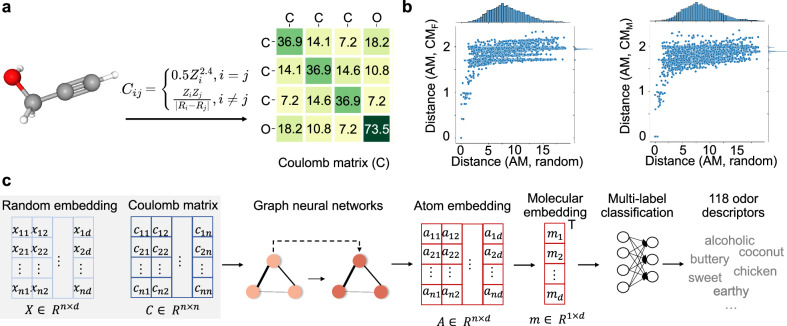
The motivation and workflow of modeling the Coulomb matrix. **a** An example of the Coulomb matrix as a fully connected graph for Propargyl alcohol, a clear colorless liquid with a geranium-like odor. Molecule’s 3D structure image can be downloaded from PubChem. **b** Similarity between Coulomb matrix and adjacency matrix, indicated by the distances. The distance is calculated with the Frobenius norm. **c** The workflow of modeling the Coulomb matrix by graph neural networks.

We calculate CM, which models atomic energies with the internuclear Coulomb repulsion operator^21,35^, and use it as our molecular representation (Supplementary Notes 1.1 and 1.2). In CM, the diagonal entries refer to a polynomial fit of atomic energies and off-diagonal entries represent the Coulomb repulsion force between atomic nuclei. Although the adjacency matrix has been widely used in molecular modeling^36,37^, CM as an emerging molecular representation can have at least two advantages: (1) CM handles the oversquashing plight by allowing direct paths between distant nodes in the fully connected graph representation (Fig. 3a); (2) distance by Frobenius norm between CM and adjacency matrix is 5–10 times smaller than that between random initialized matrix and adjacency matrix, indicating that CM is fully connected while preserving a similarity to adjacency matrix (Fig. 3b).

We build a nonlinear map (named Coulomb-GCN) between molecular structures and human olfactory perception (Fig. 3c) by replacing the adjacency matrix in message passing of GCN with CM. Specifically, starting from random atom embedding, the learned atom embedding is obtained by message passing on fully connected molecular graph with neighbor weights specified by the entries of CM. The molecular embedding is extracted by sum-pooling and fed to a multilabel classification module to predict 118 odor descriptors. Taking into account the gap between maximal and minimal entries in CM, normalization of entries may affect performance. We test Minmax and Frobenius normalizations in a matrix-wise manner.

We evaluated and compared the prediction accuracy of GCN with the adjacency matrix and those of Coulomb-GCN with the different normalizations of CM (Table 1). Compared to the GCN with adjacency matrix (AUROC of 0.678), gains in AUROC are observed in Coulomb-GCN with Frobenius normalization (AUROC of 0.759) and min–max normalization (AUROC of 0.713). Coulomb-GCN with Frobenius normalization also achieves higher performances in five out of six evaluation metrics (Table 1): AUROC (improved from 0.678 to 0.759), AUPRC (improved from 0.111 to 0.143), specificity (improved from 0.625 to 0.744), precision (improved from 0.079 to 0.089), and accuracy (improved from 0.726 to 0.780).

**Table 1:** Prediction performances of Coulomb matrix with Minmax and Frobenius normalizations and adjacency matrix in GCN

Representation	Normalization^a^	AUROC	AUPRC	Precision	Recall	Specificity	Accuracy
Adjacency matrix	–	0.678	0.111	0.079	**0.827**	0.625	0.726
Coulomb matrix	Minmax	0.713	0.138	0.082	0.811	0.687	0.749
Coulomb matrix	Frobenius	**0.759**	**0.143**	**0.089**	0.816	**0.744**	**0.780**

The highest score of each metric is shown in bold.

^a^Normalization refers to the normalization methods for the Coulomb matrix.

### Positional encoding by Laplacian eigenfunctions improves prediction accuracy

The graph Laplacian and its spectral information enable us to characterize the global and substructures of graphs^38–40^. Specifically, the graph Laplacian is defined as *L* = *D* − *A*, where *D* and *A* refer to the degree and adjacency matrices. *L* is positive semi-definite with one trivial and the other nontrivial eigenvalues. In this work, the Laplacian defined by CM acts as an extension of the normal Laplacian (*L* = *D* − *X*), where *X* refers to the weighted matrix (CM in this work) and possesses the same properties as the graph Laplacian (e.g., symmetric and positive semi-definite). In particular, the eigenvectors of *L* provide an optimal solution to the Laplacian quadratic form (${f}^{T}Lf=1/2{\sum }_{i,j}X(i,j){({f}_{i}-{f}_{j})}^{2}$)^38–40^, encoding the geometric information of graphs.

**Fig. 4:**
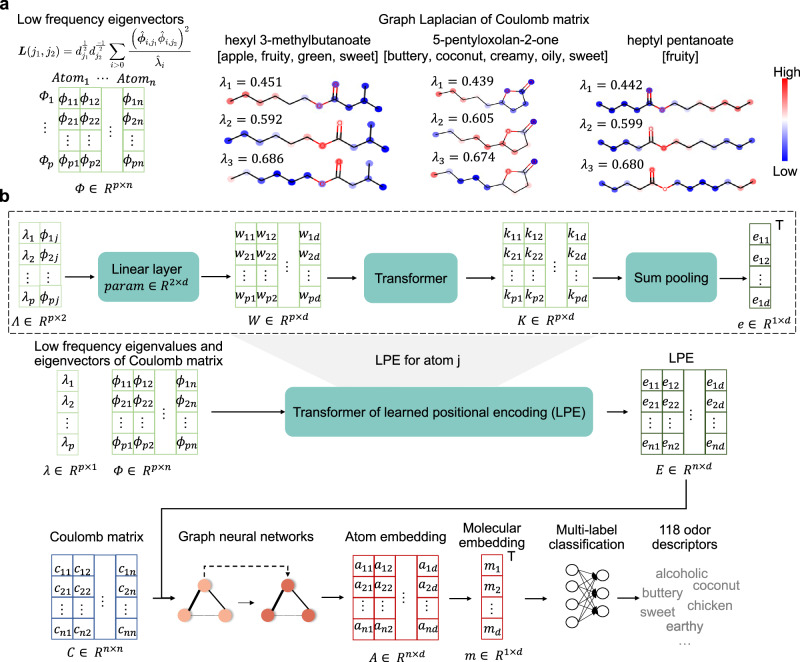
The motivation and architecture of Mol-PECO. **a** Structural information carried by the Laplace spectrum of the Coulomb matrix. Low-frequency eigenvectors, calculated with graph Laplacian, as the input matrix for positional encoding and three examples, including cyclic and acyclic molecules, of eigenvalue *λ*_*i*_ and eigenvector *ϕ*_*i*_ for molecular graphs (*i* ∈ {1, 2, 3}). The color indicates the value of each component (node) of the eigenvectors. **b** The architecture of Mol-PECO. Mol-PECO learns the positional encoding (LPE) with Transformer of graph Laplacian, and updates the atom embedding with GCN of Coulomb matrix and LPE. Specifically, GCN is implemented with skip-connection to relieve oversmoothing. Coulomb matrix, the fully connected graph representation, suppresses oversquashing with direct connections between nodes. With the updated atom embedding, Mol-PECO extracts the molecular embedding with sum-pooling, and predicts 118 odor descriptors with neural networks of molecular embedding. In this work, *p* and *d* are set as 20 and 32, respectively.

Given these properties of the Laplacian graph, we used the Laplacian eigenfunctions of the CM to encode the positional information of molecular graphs. Typical results of a cyclic odorant (5-pentyloxolan-2-one, flowing from left to right in *λ*_1_) and acyclic odorants (hexyl 3-methylbutanoate, flowing from left to right in *λ*_1_, and heptyl pentanoate, flowing from right to left in *λ*_1_) demonstrate the information carried by low-frequency eigenfunctions (Fig. 4a). Combining it with the Coulomb-GCN, we construct the deep learning framework, named Mol-PECO (Fig. 4b), with the fully connected molecular representation by CM and the positional encoding by Laplacian. We introduce a learned positional encoding (LPE) by Transformer^17^ to build the atom embedding (Supplementary Note 2.1). Specifically, LPE starts with concatenating the *p* lowest eigenvalues and the corresponding eigenvectors as the input matrix Λ ∈ *R*^*p*×2^, and learns the encoding with Transformer for every atom^17^. We obtain AUROC of 0.796 and AUPRC of 0.153 with LPE of raw CM. We further perform the experiments for LPE of asymmetric normalized CM and obtain an additional gain of performances by 0.017 and 0.028 for AUROC and AUPRC, respectively.

We compare Mol-PECO with the baseline models (Table 2): the conventional GCN of graph representations, including the adjacency matrix and the CM, and the classifiers of fingerprint representations, including Mordreds features (mordreds)^9^, bit-based fingerprints (bfps)^8^, and count-based fingerprints (cfps)^8^. Conventional classifiers include *k*-Nearest Neighbor (KNN), random forest (RF), and gradient boosting (GB). In the fingerprint methods, we first handle the problem of imbalanced label distribution with Synthetic Minority Over-sampling Technique (SMOTE)^41^, and then perform the classification. Mol-PECO outperforms the baselines in three out of six evaluation metrics (Table 2), with AUROC improved from 0.761 (cfps-KNN) to 0.813, AUPRC improved from 0.144 (mordreds-RF) to 0.181, and accuracy improved from 0.780 (Coulomb-GCN) to 0.808. In particular, Mol-PECO can balance AUROC (0.813) and AUPRC (0.181) whereas the ML method (cfps-KNN) with the highest AUROC of 0.761 has a low AUPRC of 0.057 and one (cfps-RF) with the highest AUPRC of 0.144 shows a low AUROC of 0.723. Moreover, Mol-PECO also shows superior performances of AUROC and AUPRC, compared with a recently published graph convolution model (Supplementary Note 2.4). Thus, Mol-PECO boosts the predictability of QSOR.

**Table 2:** Prediction performances by conventional classify with ML methods, GCN with adjacency and Coulomb matrices, and Mol-PECO

Baseline^a^	AUROC^b^	AUPRC^b^	Precision^b^	Recall^b^	Specificity^b^	Accuracy^b^
cfps-KNN	0.761	0.057	0.065	0.760	0.764	0.762
bfps-KNN	0.758	0.055	0.064	0.748	0.769	0.759
mordreds-KNN	0.729	0.052	0.062	0.676	0.783	0.730
mordreds-RF	0.723	0.144	0.241	0.483	**0.964**	0.723
cfps-RF	0.689	0.137	**0.258**	0.418	0.961	0.690
bfps-RF	0.671	0.119	0.227	0.381	0.962	0.672
mordreds-GB	0.725	0.126	0.220	0.499	0.951	0.725
cfps-GB	0.701	0.120	0.210	0.453	0.950	0.702
bfps-GB	0.687	0.111	0.193	0.428	0.948	0.688
adjacency-GCN	0.678	0.111	0.079	**0.827**	0.625	0.726
Coulomb-GCN	0.759	0.143	0.089	0.816	0.744	0.780
Mol-PECO-sym	0.796	0.153	0.088	0.817	0.787	0.802
Mol-PECO-asym	**0.813**	**0.181**	0.104	0.819	0.797	**0.808**

The highest and the second highest scores of each metric are shown in bold and underlined, respectively.

^a^Baseline includes conventional classifiers of fingerprint representations and graph convolutional networks (GCN) of molecular graphs. Fingerprint representations include count-based fingerprints (cfps), bit-based fingerprints (bfps), and Mordreds features (mordreds). The conventional classifiers include *k*-nearest Neighbor (KNN), random forest (RF), and gradient boosting (GB). Molecular graph representations include adjacency matrix (adjacency-GCN) and Coulomb matrix (Coulomb-GCN). Mol-PECO-sym refers to the performances with LPE of raw Coulomb matrix. Mol-PECO-asym refers to the performances with LPE of asymmetrically normalized Coulomb matrix.

^b^The evaluation metrics are calculated in testing set.

### Prediction accuracy and predictability of individual descriptors

**Fig. 5:**
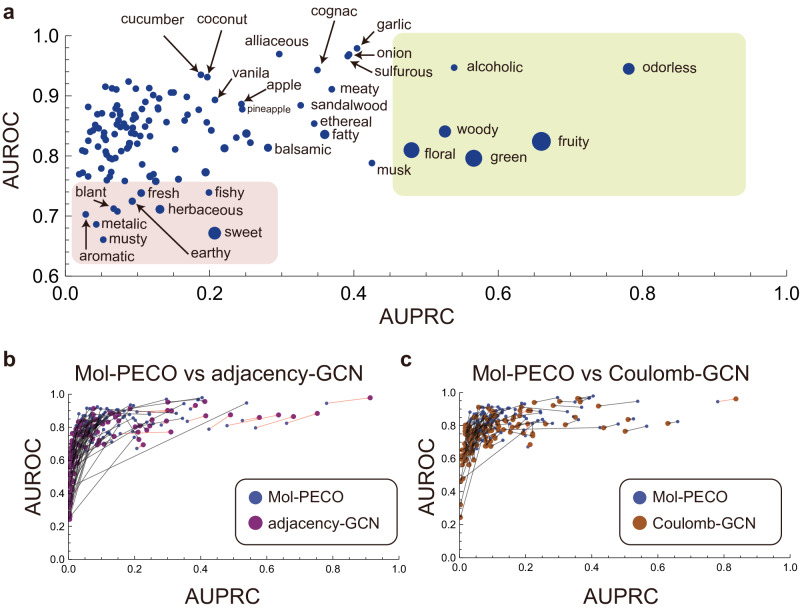
The scores of individual descriptors obtained by Mol-PECO, adjacency-GCN, and Coulomb-GCN. **a** The scores of individual descriptors obtained by Mol-PECO. Each point corresponds to the score (AUPRC, AUROC) for each descriptor. The diameter of points is proportional to the square root of the frequency of the descriptor in the dataset. The yellow region indicates highly predictable descriptors, whereas the red region accommodates descriptors that are difficult to predict from chemical structures by Mol-PECO. **b**, **c** Comparisons of scores obtained by Mol-PECO (blue points) with those by (**b**) adjacency-GCN (purple points) and by (**c**) Coulomb-GCN. Each arrow links the score of Mol-PECO with that of adjacency-GCN or Coulomb-GCN for each descriptor. Arrows are colored in red if both AUPRC and AUROC of Mol-PECO are lower than those of adjacency-GCN or Coulomb-GCN.

We scrutinize the prediction obtained by Mol-PECO by computing the AUROC and AUPRC for each descriptor and contrasting the results with other GCN models (Supplementary Note 2.2). Figure 5a shows the scores of individual descriptor obtained by Mol-PECO. Descriptors exhibiting high performance, as demarcated by the yellow region, predominantly include common descriptors such as “odorless”, “fruity”, “floral”, “green”, and “woody”, with the exception of “alcoholic”.

Mol-PECO’s average performance enhancement is ascribed to its capacity to augment the scores across a broad spectrum of infrequent descriptors, thereby demonstrating its versatility. This is in stark contrast to adjacency-GCN, which is limited to predicting only common descriptors (Fig. 5b). The limitations of adjacency-GCN are somewhat ameliorated in Coulomb-GCN (Fig. 5c), thereby supporting the hypothesis that oversquishing hampers efficient training of conventional GCNs. Additionally, the observed decrease in Mol-PECO’s performance for common descriptors underscores a trade-off of prediction accuracy between a limited set of frequent descriptors and a diverse array of infrequent ones.

Moreover, Mol-PECO identifies descriptors challenging to predict only from chemical structures, as illustrated by the red region in Fig. 5a. These descriptors are categorizable into three classes: (1) Descriptors pertaining to senses other than olfaction, such as “sweet” (taste), “creamy” (touch), “metallic” (touch, temperature); (2) Conceptual and polysemous descriptors including “bland”, “fresh”, “earthy”, “aromatic”; (3) Categorically complex descriptors like “fishy”, “musty”, “herbaceous”, “herbal”, “cortex”.

The first class of descriptors may be perceived as olfactory properties due to the associative learning of olfaction with other sensory modalities. Consequently, predicting these descriptors solely from chemical structures poses significant difficulties or may even be impossible. The second category encompasses conceptual descriptors such as “bland”, indicative of potentially combinatorial attributes in odorant mixtures. The third category, while more specific than the second, pertains to descriptors delineating complex entities like fish, mold, and skin. Given the abstract nature of the second and third categories, their accurate prediction may necessitate extensive datasets, akin to the approach employed in large language model training.

### External test with DREAM dataset

To further investigate Mol-PECO’s generalization ability, we further assess the trained Mol-PECO’s performances in an external test dataset, DREAM dataset. The DREAM project measures 49 volunteers’ perception score (from 0 to 100) of molecules in 2 dilutions (low and high concentration). After transforming the regression annotations into binary labels (Supplementary Note 2.3), we obtain the final DREAM test set: 13 molecules with 2 odor descriptors in the low concentration set and 31 molecules with 3 odor descriptors in the high concentration.

We assess Mol-PECO with the DREAM dataset in high/low concentration independently. For low concentration, Mol-PECO achieves perfect performances (AUROC/AUPRC of 1.00/1.00), and all “sweet”/”garlic” molecules are correctly distinguished from the rest (Table 3). For high concentration, the “burnt” molecule has been correctly predicted with AUROC of 1.00 (Table 3), while “fruity” and “sweet” molecules achieve AUROC of 0.88 and 0.60 (Table 3), respectively.

**Table 3:** Performances of Mol-PECO in the external DREAM dataset

Concentration	Odor descriptor	AUROC	AUPRC	Precision	Recall	Specificity	Accuracy
Low	Garlic	1.000	1.000	1.000	1.000	1.000	1.000
Low	Sweet	1.000	1.000	1.000	1.000	1.000	1.000
High	Burnt	1.000	1.000	1.000	1.000	1.000	1.000
High	Fruity	0.880	0.457	0.400	1.000	0.778	0.889
High	Sweet	0.600	0.984	1.000	0.600	1.000	0.800

### Learned odor space by Mol-PECO

To elucidate the structural organization of multiple odors in relation to descriptors, we conducted a dimensionality reduction for the output of Mol-PECO’s penultimate layer to construct a latent odor space, which was then evaluated at both global and local scales. Globally, the analysis focused on assessing the extent to which clusters of odors within this latent space encapsulate descriptor information. Locally, the investigation centered on determining whether individual molecules are characterized by a set of odor descriptors that are similar to those of proximal molecules.

**Fig. 6:**
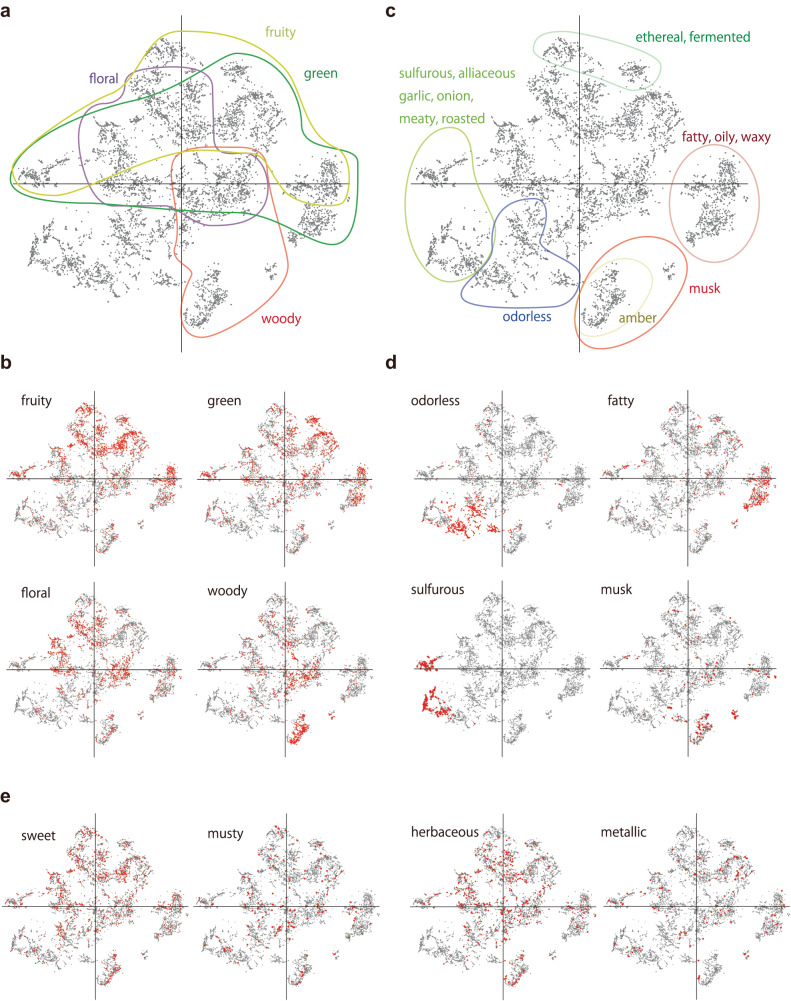
The odor space built from Mol-PECO and its global property obtained by dimensionality reduction with t-SNE. Each gray dot corresponds to an individual molecule. **a** The global distribution of molecules, highlighting those associated with the representative common descriptors, “fruity”, “green”, “floral”, and “woody”. **b** The distribution of molecules corresponding to each descriptor in (**a**); the molecules to which the descriptor is assigned are marked in red, and those to which it is not assigned are marked in gray. **c** The global distributions of molecules associated with the descriptors that form distinct clusters. **d** The distribution of molecules corresponding to a selected descriptor representative of each cluster identified in (**c**). **e** The distributions of molecules associated with descriptors with low prediction scores by Mol-PECO: “sweet”, “musty”, “herbaceous”, and “metallic”.

For global structure, Fig. 6a, b illustrates the distribution patterns of the frequent descriptors, “fruity”, “floral”, “green”, “woody”, which are among the top six most frequently assigned descriptors in the dataset. Molecules with these descriptors exhibit dispersed distributions across the odor space, yet distinct regions can be identified as the overlapping of some descriptors. On the periphery of the space, more defined clusters are formed by specific classes of descriptors, such as “odorless”, “fatty”, “sulfurous”, “ethereal”, and “musk”, as shown in Fig. 6c and d (Supplementary Note 2.5 for the descriptors not shown in Fig. 6d). These descriptors are associated with high scores by Mol-PECO.

Moreover, in the context of structure-correlated descriptors, molecules linked with “alliaceous”, “garlic”, or “onion” congregate within the “sulfurous” cluster (Fig. 6c). This cluster aligns with prior research on the olfactory characteristics of sulfur-containing compounds^42,43^. In the case of synonymy descriptors, molecules associated with “oily” and “waxy” are observed to cluster with “fatty”, corroborating their semantic similarity (Fig. 6c). These clustering patterns visually substantiate the efficient representation of odorant molecules within the learned odor space by Mol-PECO, which was quantified by its enhanced performance metrics in Fig. 5.

Conversely, molecules associated with lower-scored descriptors such as “sweet”, “musty”, “herbaceous”, and “metallic” are distributed across the space without discernible pattern (Fig. 6e). This uniform distribution underscores the difficulty in representing these descriptors within the learned space. Finally, it should be noted that the dimensionality reduction of the odor space to two dimensions via principal component analysis (PCA) does not reveal specific patterns or structures. This observation means that the learned odor space possesses a high-dimensional and nonlinear structure to accommodate molecules with a wide array of structural characteristics. Thus, the two-dimensional projection by t-SNE delineates only the limited aspects of the learned odor space.

**Fig. 7:**
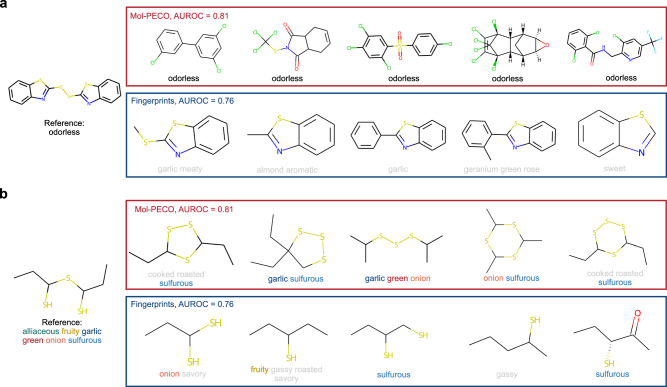
Local view of the learned odor space investigated with nearest neighbor retrieval. **a** Retrieved molecules for an odorless molecule by Mol-PECO and the best fingerprints method. **b** Retrieved molecules for an odorant molecule by Mol-PECO and the best fingerprints method.

For local structure, we investigate one odorless molecule (triphenylphosphane) and one odorant molecule (1-(1-sulfanylpropylsulfanyl)propane-1-thiol with descriptors of “alliaceous”, “fruity”, “garlic”, “green”, “onion”, and “sulfurous”) as the examples (Fig. 7a, b). We compare the top-5 nearest molecules searched by Mol-PECO’s embedding and bfps. The top-5 molecules of Mol-PECO and bfps are calculated with cosine similarity and Tanimoto similarity, respectively. For the odorless molecule, Mol-PECO retrieves all neighbors with the odorless descriptor, whereas bfps retrieves no molecule. Notably, the molecules fetched by Mol-PECO possess different substructures compared with the reference (e.g., all with C-Cl bond and top-2/3/5 with carbonyl functional group). For the odorant molecule, Mol-PECO retrieves all neighbors with shared descriptor, and bfps retrieves four. Moreover, all of the molecules retrieved by bfps are open-chain structured, the same with the reference. In contrast, Mol-PECO retrieves quite different structured molecules, four of which are cyclic molecules. Both examples would indicate Mol-PECO’s promising potential in decoding molecules with different structures but identical smells. Nevertheless, such generalization capacity of structural features within the current framework still has room for improvement. This limitation is illustrated in the categorization of molecules possessing a musk-like aroma. This category encompasses molecules that possess disparate functional groups yet share an identical smell. For musk-scent molecules, Mol-PECO demonstrates a propensity to assign high scores to macrocyclic musks, whereas it exhibits a deficiency in identifying nitro-musk compounds as musk-scented molecules (Supplementary Note 2.6). This discrepancy underscores a critical area for improvement in the algorithm’s generalization ability across varied structural features while maintaining accurate scent classification.

## Discussion

In this work, we develop Mol-PECO for predicting human olfactory perception from molecular structures. We handle this QSOR problem by improving the graph neural network-based approach in two aspects: the molecular representation and the graph modeling method. For molecular representation, we use CM, which is fully connected and contains 3D conformer information (3D coordinates and charges of atoms), instead of an adjacency matrix. For graph modeling, we use the positional encoding by the eigenfunctions of Laplacian to mitigate GCN’s deficiency in accomodating directional information of graphs. Mol-PECO improves the olfactory perception prediction in two stages; the performance raises from adjacency-GCN (AUROC of 0.678) to Coulomb-GCN (AUROC of 0.759) by introduction of CM, followed by from Coulomb-GCN (AUROC of 0.759) to Mol-PECO with eigenfunctions of the symmetric (AUROC of 0.796) and asymmetric Laplacian (AUROC of 0.813). These gains in average performance are achieved by the enhanced capability of Mol-PECO to predict diverse infrequent descriptors. The latent odor space learned by Mol-PECO captures coherent global and local relationships by coordinating the structural and perceptual information of molecules.

These results indicate that facilitation and acceleration of training and also improvement of the expressive power of GCN can greatly contribute to the learning of nontrivial relationships between molecular structures and olfactory descriptors (labels) in the QSOR problem. Although extensions of NN architectures are typically accompanied by an increased cost of learning, the limited size of odorant molecules up to 400 daltons in molecular weight enables the practical application of such extended architectures. Thus, we believe that the QSOR problem provides a good real-world task to test and demonstrate the effectiveness of advanced architectures. In particular, pre-training of graph representation using unlabeled data may lead to further improvements in the accuracy of QSOR prediction^44,45^. In addition, as we found by experiments, the employment of asymmetric Laplacians might also contribute to further technical advancements.

Nevertheless, the QSOR problem suffers from ambiguity in the labels (descriptors) assigned to each molecule and also from low objectivity and consistency of the labeling conducted by few specialists^46,47^. In particular, individuals can have a quite different sense to highly ambiguous descriptors such as “sweet”^47^. Thus, consistent labeling by individuals requires a certain amount of training, resulting in the difficulty to increase the amount of data. Furthermore, for the prediction of mixed odors, which is important for applications, data acquisition is prohibitive due to the huge number of possible combinations^48^. In the future, it will be important to apply the approach developed in this paper to the prediction of more objective and systematically measurable outputs, such as the chemical properties of odorants^49^, the response of olfactory receptors^50,51^, and the response of neural activities^2,52^. Such extensions will lead to more comprehensive and data-oriented understanding of chemical information coding in the olfactory system.

## Methods

### Coulomb matrix and its normalization

As a molecular representation, Coulomb matrix is calculated mainly based on Coulomb replusion force as follows:

1 $${C}_{ij}=\left\{\begin{array}{ll}0.5{Z}_{i}^{2.4}\quad \,\,{{\mbox{for}}}\,i=j\\ \frac{{Z}_{i}{Z}_{j}}{\left\vert {{{{\bf{R}}}}}_{i}-{{{{\bf{R}}}}}_{j}\right\vert }\quad \,\,{{\mbox{for}}}\,i\,\ne\, j\end{array}\right.,$$

where *C*_*ij*_ refers to the entry in *i*th row and *j*th column, *Z*_*i*_ refers to the atomic charge, and *R*_*i*_ refers to the relative coordinates.

Considering the gap of minimal and maximal entries in a Coulomb matrix, we performed 2 preprocessing methods to handle it, including matrix-wised Frobenius normalization and min–max normalization as follows:

2 $$\begin{array}{rcl}| | C| {| }_{F}&=&\sqrt{\sum\limits_{i=1}^{n}\sum\limits_{j=1}^{n}{C}_{ij}^{2}},\\ {C}_{max}&=&\max\limits_{i,j}{C}_{ij},\\ {C}_{min}&=&\min\limits_{i,j}{C}_{ij},\\ \end{array}$$

where ∣∣*C*∣∣_*F*_ refers to the normalization term of Frobenius normalization, *C*_*max*_ and *C*_*min*_ refer to the max and min term in min–max normalization. With the normalization terms, we calculated the preprocessed Coulomb matrix as follows:

3 $$\begin{array}{rcl}{C}^{F}&=&C/(| | C| {| }_{F}+\epsilon ),\\ {C}^{M}&=&(C-{C}_{min})/({C}_{max}-{C}_{min}+\epsilon ),\\ \end{array}$$

where *C*^*F*^ and *C*^*M*^ refer to the Frobenuis- and minmax- normalized matrices, and *ϵ* equals to 10^−9^.

### GCN of Coulomb matrix for multilabel classification

We build Coulomb-GCN based on Coulomb matrix with three modules: (1) atom embedding updating, (2) molecular embedding extraction, and (3) multilabel classification. In atom embedding updating, we use GCN with the residual mechanism to learn the molecular embedding as follows:

4 $${H}^{(l)}=\sigma \left(X{H}^{(l-1)}{W}_{graph}^{(l-1)}\right)+{H}^{(l-1)}{W}_{linear}^{(l-1)},$$

where *H*^(*l*)^ ∈ *R*^*n*×*d*^ refers to the updated atom embedding in *l*th layer, *X* ∈ *R*^*n*×*n*^ refers to Coulomb matrix, *H*^(*l*−1)^ ∈ *R*^*n*×*h*^ refers to the updated atom embedding in *l* − 1th layer, ${W}_{graph}^{(l-1)}\in {R}^{h\times d}$ refers to the parameters in GCN, ${W}_{linear}^{(l-1)}\in {R}^{h\times d}$ refers to the parameters in the linear transformation of *H*^(*l*−1)^ for the residual mechanism, *H*^(0)^ refers to the randomly initialized atom embedding, *n* refers to the number of atom in the molecule, *d* refers to the dimension of embedding in *l*th layer, *h* refer to the dimension of embedding in *l* − 1th layer, and *σ* refers to SELU activation function^53^. In molecular embedding extraction, we use the sum-pooling function as follows:

5 $${m}_{i}=\sum\limits_{j\in [n]}{H}_{ji}^{(l)},$$

where *m*_*i*_ refers to the *i*th entry in the molecular embedding of molecule *m*. In multilabel classification, we use fully connected layers as follows:

6 $$y=\sigma (m{W}_{clf}),$$

where *m* ∈ *R*^1×*d*^ refers to the learned molecular embedding, *W*_*clf*_ ∈ *R*^*d*×*o*^ refers to the parameters in the fully connected layer, and *o* refers to the number of odor descriptors. Coulomb matrix and Coulomb-GCN have been implemented by Python (version 3.7.4) with deepchem (version 2.6.1) and pytorch (version 1.12.1).

### Positional encoding by Laplacian for multilabel classification

Mol-PECO differs from Coulomb-GCN only in the atom embedding updating module, where we build the learned positional encoding (LPE).

The Laplacian matrix (*L*^1^) of Coulomb matrix (*X*) is calculated as follows:

7 $${L}^{1}=D-X,$$

where *D* refers to the degree matrix. In LPE with eigendecomposition of Laplacian, symmetrical Laplacian matrix is calculated for further spectral decomposition as follows:

8 $$\begin{array}{rcl}{L}^{2}\,=\,I-{D}^{-1/2}X{D}^{-1/2}={D}^{-1/2}D{D}^{-1/2}-{D}^{-1/2}X{D}^{-1/2}\\ \,=\,{D}^{-1/2}(D-X){D}^{-1/2}={D}^{-1/2}{L}^{1}{D}^{-1/2}.\\ \end{array}$$

where *I* refers to the identity matrix. With the equations, *L*_2_ is calculated with *L*_1_ divided by $\sqrt{{d}_{ii}{d}_{jj}}$. Let ${d}_{ii}={\sum }_{j = 0}^{i-1}{a}_{ij}+\mathop{\sum }_{j = i+1}^{n}{a}_{ij}+{a}_{ii}$, then the Laplacian is calculated as follows:

9 $$\begin{array}{l}{L}_{ii}^{1}\,=\,\mathop{\sum}\limits_{j=0}^{i-1}{a}_{ij}+\mathop{\sum}\limits_{j=i+1}^{n}{a}_{ij},\\ {L}_{ij}^{1}\,=\,-{a}_{ij},i\,\ne\, j,\\ \end{array}$$

which indicates whether setting the diagonal entries zeros or not has no influence on *L*^1^ and *L*^2^ as *L*^2^ = *D*^−1/2^*L*^1^*D*^−1/2^.

With Laplacian, Mol-PECO updates the positional encoding in an atom-wise manner proposed in Spectral Attention Network^17^. Mol-PECO learns LPE in an atom-by-atom manner. Specifically, Mol-PECO first performs the linear transformation of eigenvalues and eigenvectors in a single atom, learns the positional information by Transformer, and then extracts the atom positional encoding by sum-pooling as follows:

10 $$\begin{array}{l}W\,=\,\Lambda {W}_{0},\\ K\,=\,Transformer(W),\\ {e}_{i}\,=\,\mathop{\sum}\limits_{j\in [n]}{K}_{ji},\end{array}$$

where Λ ∈ *R*^*p*×2^ refers to the *p* eigenvalues and eigenvectors for a single atom, *W*_0_ ∈ *R*^2×*d*^ refers to the parameters of the linear transformation in LPE, *K* ∈ *R*^*p*×*d*^ refers to the updated embedding with Transformer of *W* ∈ *R*^*p*×*d*^, and *e*_*i*_ refers to the *i*th entry of single atom embedding (vector-shaped representation). Graph Laplacian and Mol-PECO have been implemented by Python (version 3.7.4) with deepchem (version 2.6.1) and PyTorch (version 1.12.1).

### Loss functions

For both Coulomb-GCN and Mol-PECO, we build the classification loss with the binary cross-entropy loss function and a logarithm regularization term as follows:

11 $$\begin{array}{l}{l}^{i}({y}_{true}^{i},{y}_{pred}^{i})\,=\,BCE({y}_{true}^{i},{y}_{pred}^{i})+| \log ({y}_{true}^{i}+\epsilon )-\log ({y}_{pred}^{i}+\epsilon )| ,\\ l({y}_{true},{y}_{pred})\,=\,\displaystyle\frac{1}{o}\mathop{\sum}\limits_{i\in [o]}{w}_{i}{l}^{i}({y}_{true}^{i},{y}_{pred}^{i}),\end{array}$$

where ${y}_{true}^{i}$ and ${y}_{pred}^{i}$ refer to the ground truths and predictions of *i*th odor descriptor, *l*^*i*^ refers to the loss function of *i*th odor descriptor, BCE refers to the binary cross-entropy function, *w*_*i*_ refers to $1-{n}_{pos}^{i}/{n}_{tot}$, ${n}_{pos}^{i}$ refers to the number of positive samples in *i*th descriptor, *n*_*tot*_ refers to the number of total samples. The training process has been implemented by Python (version 3.7.4) with PyTorch (version 1.12.1).

### Molecular descriptors and graph-based representations

We use three classical molecular descriptors in this work as the baseline molecular representations, including Mordred features (mordred)^9^, bit-based Morgan fingerprints (bfps)^8^, and count-based Morgan fingerprints (cfps)^8^. Mordred calculates 2D and 3D features to construct the molecular representations. Both bfps and cfps encode the molecule’s topological environments (molecular fragments), which indicate the presence of atoms and functional groups, into a vector. Specifically, bfps encodes the presence or absence of the molecular fragments as binary information, while cfps encodes the number of atom/functional groups in the topological environment. The calculation of molecular descriptors has been implemented by Python (version 3.7.4) with mordred (version 1.2.0) and rdkit-pypi (version 2022.3.4).

We utilize the adjacency matrix as the molecular representation for training baseline GCN model. In the adjacency matrix, atoms are encoded as nodes, and bonds are encoded as edges^7^. In this way, we obtain the 2D graph representation of molecules.

### Machine learning of molecular descriptors

Machine learning of molecular descriptors used in this work as baselines include (1) Synthetic Minority Over-sampling Technique (SMOTE)^41^ to handle the imbalanced label distribution, and (2) multiple binary classifiers to predict multiple odor descriptors. For SMOTE, it performs minority sampling by generating new minority instances to expand the number of minority classes. For binary classifiers, we use *K*-Nearest Neighbor classifier (KNN), random forest classifier (RF), and gradient boosting classifier (GB). The KNN acts as a non-parametric classifier and predicts the label by voting from neighbors. RF acts as the ensemble learning of decision trees with sample bagging to decrease the variance of model and feature bagging to decrease the correlation among decision trees. GB builds multiple weak learners to minimize the differences between the true label and the predicted value by performing gradient descent. The procedures have been implemented by Python (version 3.7.4) with imblearn (version 0.9.0) and scikit-learn (version 1.0.2).

### Supplementary information

Supplemental material

## Data Availability

All datasets in this work are publicly available via GitHub (https://github.com/Q-bio-at-IIS/Mol-PECO).

## Code availability

All codes in this work are publicly available via GitHub (https://github.com/Q-bio-at-IIS/Mol-PECO).
