# Supplementary material for: A deep position-encoding model for predicting olfactory perception from molecular structures and electrostatics
Source: NPJ Syst Biol Appl. 2024 Jul 17;10:76. doi: 10.1038/s41540-024-00401-0 (PMC11255234; doi:10.1038/s41540-024-00401-0)
Supplement: Supplementary file 1 — Supplemental material [file 41540_2024_401_MOESM1_ESM.pdf]

# **Supplementary information** for Mol-PECO: a deep learning model to predict human olfactory perception from molecular structures

Mengji Zhang[1,2*], Yusuke Hiki[3], Akira Funahashi[3], Tetsuya J. Kobayashi[2*]

[1]School of Biomedical Engineering, Shanghai Jiao Tong University, Shanghai, 200030, China.
[2]Institute of Industrial Science, The University of Tokyo, Meguro-ku, Tokyo, 153-8505, Japan.
[3]Department of Biosciences and Informatics, Keio University, Kouhoku-ku, Yokohama, 223-8522, Japan.

*Corresponding author(s). E-mail(s): mengji.zhang0809@gmail.com; tetsuya@mail.crmind.net;

## 1 Supplementary Note 1

### 1.1 Molecular representations

We utilize molecular descriptors for conventional machine learning-based methods and graph-based representations for deep learning-based methods (Supplementary Figure 1). Conventional descriptors, including bit-based fingerprints, count-based fingerprints, and Mordred descriptors, transform molecules into fixed-length vectors by hand-crafted rules. Graph-based representations, including adjacency matrix and Coulomb matrix, encode the connectivity among atoms into a graph and generate the final vectors by end-to-end learning.

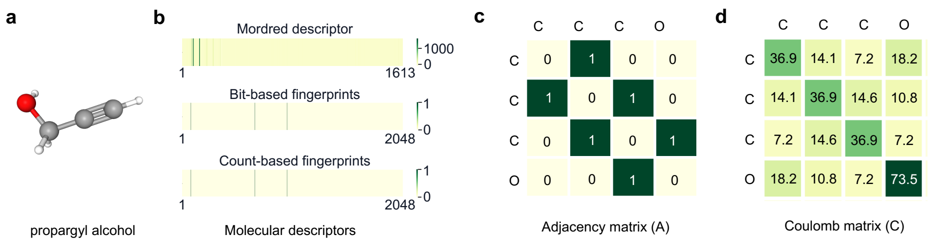


**Supplementary Figure 1** Demonstration of different molecular representations for propargyl alcohol. a) The 3D conformer structure. b) The calculated conventional molecular descriptors, including Mordred descriptor, bit-based fingperprints, and count-based fingerprints. c) The adjacency matrix. d) The Coulomb matrix.

## 1.2 Coulomb matrix for enantiomer molecules

We utilize the isomeric SMILES, from which different Coulomb matrices are calculated, to represent molecules including enantiomer molecules. In isomeric SMILES, R isomers with atoms arranged in a clockwise manner are represented by "@@", while S isomers in an anticlockwise manner are represented by "@" (Supplementary Figure 2). Additionally, clockwise and anticlockwise manners encode the differences in isomers' 3D coordinates. Thus, we have calculated different Coulomb matrices with isomeric SMILES. An enantiomer example of carvone is demonstrated in Supplementary Figure 2.

# 2 Supplementary Note 2

## 2.1 Parameter tuning of Mol-PECO

We optimize Mol-PECO with different number of Transformer layers in LPE and choose 4 as the optimized parameter (Supplementary Table 1).

**Supplementary Table 1** Scores of Mol-PECO with different number of Transformer layers and the layer number optimization. The highest scores are shown in bold.

| #Transformer layers | AUROC[1] | AUPRC[1] | Precision[1] | Recall[1] | Specificity[1] | Accuracy[1] |
|---|---|---|---|---|---|---|
| 1 | 0.809 | 0.170 | 0.103 | 0.826 | 0.789 | 0.807 |
| 2 | 0.798 | 0.147 | 0.100 | 0.817 | 0.779 | 0.798 |
| 3 | 0.807 | 0.163 | 0.090 | **0.831** | 0.779 | 0.805 |
| 4 | **0.813** | **0.181** | **0.104** | 0.819 | **0.797** | **0.808** |
| 5 | 0.807 | **0.181** | 0.089 | 0.819 | 0.780 | 0.800 |
| 6 | 0.802 | 0.162 | 0.093 | 0.814 | 0.790 | 0.802 |

[1]The evaluation metrics are calculated with the validation set.

The training process of the optimized parameter (the number of Transformer layer = 4) is monitored by the loss curves (Supplementary Figure 3a). The decreased trends of the loss curves are consistent, indicating that Mol-PECO has found the right bias/-variance tradeoff in the training and validation sets. Mol-PECO chooses the checkpoint

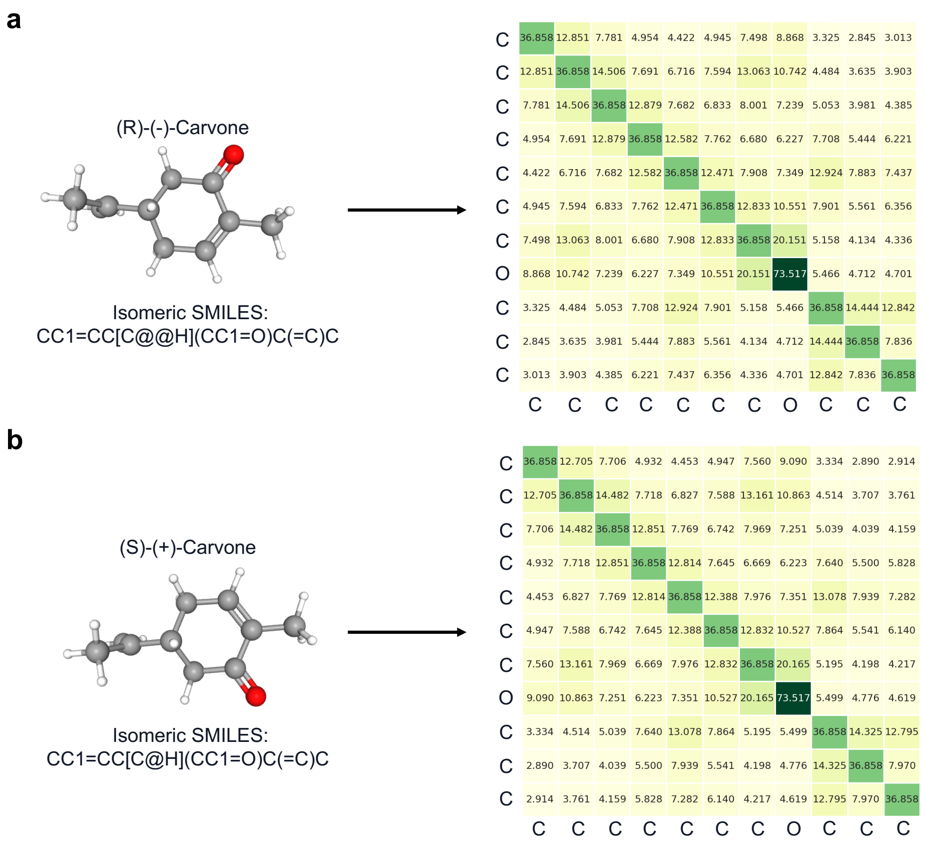


**Supplementary Figure 2** The molecular representations for carvone. a) The 3D conformer structure and Coulomb matrix for S-(+)-Carvone. b) The 3D conformer structure and Coulomb matrix for R-(-)-Carvone.

of 554 epochs with minimal loss of 0.140 as the final model for testing (Supplementary Figure 3b).

## 2.2 AUROC and AUPRC of each descriptor

After training and validation, we obtain detailed performances of 118 odor descriptors in 6 evaluation metrics (Supplementary Figure 4). AUROC and AUPRC of each descriptor obtained by Mol-PECO are listed on Supplementary Table 2.

## 2.3 External DREAM dataset and the evaluation

The DREAM dataset has 338 molecules with 19 odor descriptors, which are annotated by 49 volunteers. The molecules are labeled in two dilutions (high / low concentration). This means that a molecule is annotated with 49 scores (ranging from 0 to 100) by 49 volunteers. For example, 4-Hydroxybenzaldehyde has 49 different scores for

**Supplementary Table 2** AUROC and AUPRC of each descriptor obtained by Mol-PECO. In each box, the top, middle, and bottom items are descriptor, AUROC, and AUPRC, respectively.

| | | | | | | | |
|---|---|---|---|---|---|---|---|
| alcoholic | aldehydic | alliaceous | almond | amber | ambre | animal | anisic |
| 0.947 | 0.869 | 0.97 | 0.809 | 0.877 | 0.846 | 0.786 | 0.854 |
| 0.539 | 0.096 | 0.296 | 0.023 | 0.185 | 0.046 | 0.106 | 0.081 |
| apple | apricot | aromatic | balsamic | banana | berry | bland | brandy |
| 0.886 | 0.839 | 0.703 | 0.814 | 0.878 | 0.766 | 0.712 | 0.912 |
| 0.244 | 0.054 | 0.028 | 0.281 | 0.106 | 0.061 | 0.067 | 0.065 |
| burnt | buttery | camphor | caramel | cedar | celery | cheesy | cherry |
| 0.868 | 0.821 | 0.838 | 0.833 | 0.911 | 0.77 | 0.913 | 0.843 |
| 0.065 | 0.089 | 0.091 | 0.104 | 0.071 | 0.122 | 0.116 | 0.038 |
| chocolate | citrus | clean | cocoa | coconut | coffee | cognac | cooked |
| 0.802 | 0.813 | 0.847 | 0.861 | 0.931 | 0.912 | 0.943 | 0.915 |
| 0.057 | 0.221 | 0.084 | 0.094 | 0.197 | 0.098 | 0.349 | 0.055 |
| cortex | creamy | cucumber | dairy | dry | earthy | ethereal | fatty |
| 0.758 | 0.708 | 0.935 | 0.821 | 0.812 | 0.725 | 0.854 | 0.836 |
| 0.082 | 0.072 | 0.188 | 0.088 | 0.086 | 0.093 | 0.345 | 0.36 |
| fermented | fishy | floral | fresh | fruity | garlic | gassy | geranium |
| 0.83 | 0.739 | 0.81 | 0.738 | 0.824 | 0.979 | 0.869 | 0.836 |
| 0.237 | 0.199 | 0.48 | 0.105 | 0.66 | 0.404 | 0.076 | 0.068 |
| grape | grapefruit | grassy | green | hay | hazelnut | herbaceous | herbal |
| 0.827 | 0.878 | 0.766 | 0.796 | 0.795 | 0.874 | 0.711 | 0.758 |
| 0.056 | 0.129 | 0.031 | 0.566 | 0.065 | 0.075 | 0.131 | 0.125 |
| honey | hyacinth | jasmin | lactonic | lavender | leafy | leathery | lemon |
| 0.82 | 0.837 | 0.816 | 0.88 | 0.882 | 0.773 | 0.848 | 0.808 |
| 0.1 | 0.096 | 0.088 | 0.128 | 0.032 | 0.063 | 0.076 | 0.077 |
| lily | marine | meaty | medicinal | melon | metallic | mild | milky |
| 0.852 | 0.884 | 0.911 | 0.796 | 0.859 | 0.686 | 0.76 | 0.832 |
| 0.041 | 0.072 | 0.369 | 0.069 | 0.116 | 0.043 | 0.057 | 0.053 |
| mint | muguet | mushroom | musk | musty | natural | nut | odorless |
| 0.843 | 0.924 | 0.852 | 0.788 | 0.661 | 0.807 | 0.761 | 0.945 |
| 0.202 | 0.088 | 0.071 | 0.421 | 0.052 | 0.027 | 0.157 | 0.774 |
| oily | onion | orange | orris | ozone | patchouli | peach | pear |
| 0.773 | 0.969 | 0.797 | 0.849 | 0.859 | 0.896 | 0.901 | 0.906 |
| 0.194 | 0.393 | 0.061 | 0.11 | 0.074 | 0.13 | 0.122 | 0.15 |
| phenolic | pine | pineapple | plum | popcorn | powdery | pungent | ripe |
| 0.856 | 0.905 | 0.878 | 0.84 | 0.779 | 0.847 | 0.864 | 0.825 |
| 0.196 | 0.146 | 0.245 | 0.034 | 0.05 | 0.093 | 0.098 | 0.032 |
| roasted | rose | rum | sandalwood | savory | soapy | sour | spicy |
| 0.893 | 0.837 | 0.879 | 0.884 | 0.811 | 0.871 | 0.807 | 0.766 |
| 0.207 | 0.251 | 0.07 | 0.326 | 0.152 | 0.051 | 0.056 | 0.117 |
| strawberry | sulfurous | sweet | tea | tobacco | tropical | vanilla | vegetable |
| 0.869 | 0.966 | 0.671 | 0.77 | 0.815 | 0.822 | 0.898 | 0.856 |
| 0.044 | 0.391 | 0.207 | 0.019 | 0.045 | 0.256 | 0.168 | 0.14 |
| vetiver | violet | warm | waxy | winey | woody | | |
| 0.89 | 0.869 | 0.772 | 0.859 | 0.833 | 0.841 | | |
| 0.111 | 0.169 | 0.024 | 0.165 | 0.117 | 0.526 | | |

[1]The evaluation metrics are calculated with the validation set.

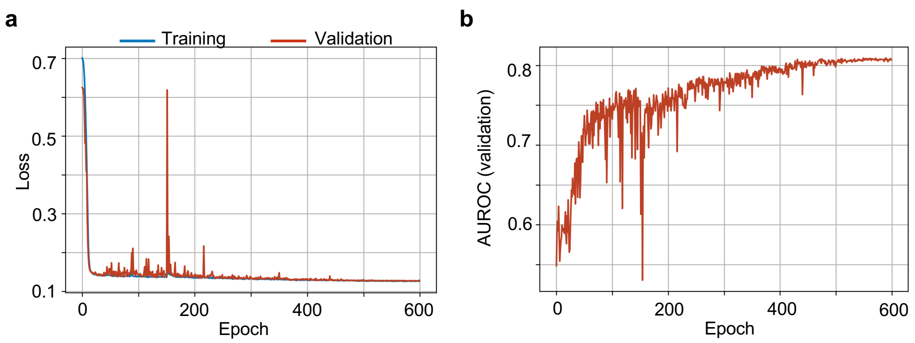


**Supplementary Figure 3** The training process with the optimized parameter. (a) The unweighted loss during training. (b) The unweighted AUROC during training.

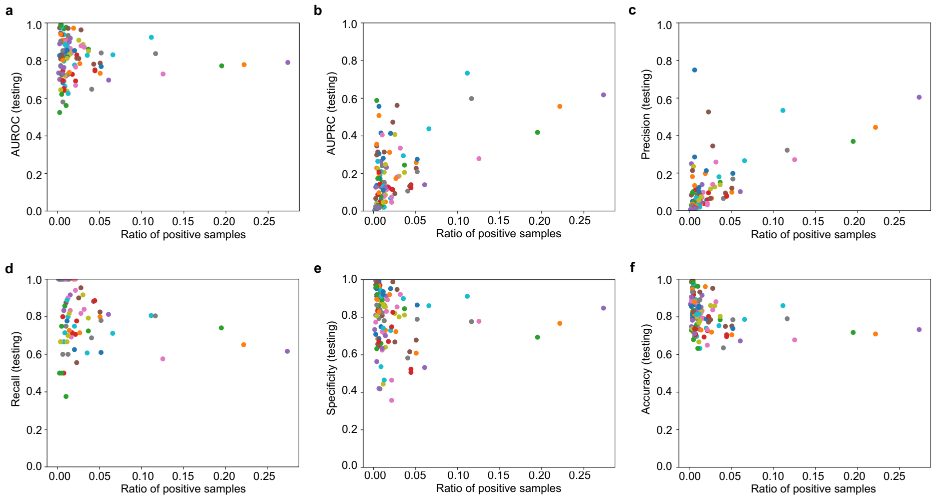


**Supplementary Figure 4** The detailed performances of Mol-PECO in 6 evaluation metrics, including (a) AUROC, (b) AUPRC, (c) Precision, (d) Recall, (e) Specificity, and (f) accuracy. The $x$-axis refers to the ratio of positive samples. Each dot refers to one odor descriptor.

the 'sweet' descriptor. Thus, the task addressed in DREAM is regression of personalized perception, whereas our Mol-PECO is designed for the classification of grouped percetion.

To transform the regression problem into a classification problem, we obtained the binary labels of odor descriptors of the DREAM dataset by majority voting and set the voting cutoff as 60%. After curating the dataset for classification, we obtained the final DREAM test set: 13 molecules with 2 odor descriptors in the low concentration set (Supplementary Table 3) and 31 molecules with 3 odor descriptors in the high concentration (Supplementary Table 4).

For low concentration, Mol-PECO achieves AUROC of 1.000 for descriptor of 'garlic' and 'sweet' (Supplementary Figure 5). For high concentration, Mol-PECO achieves

**Supplementary Table 3** The curated DREAM dataset of low concentration.

| DREAM ID | SMILES | Odor descriptor |
|---|---|---|
| 1 | COC1=C(C=CC(=C1)C=O)O | sweet |
| 2 | COC1=CC2=CC=CC=C2C=C1 | sweet |
| 3 | CC(=O)C1=CC2=CC=CC=C2C=C1 | sweet |
| 4 | CCOC(=O)CC(=O)OCC | sweet |
| 5 | CCCC(=O)OCC | sweet |
| 6 | COC(=O)C1=CC=CC=C1N | sweet |
| 7 | CCCCC(=O)OCC | sweet |
| 8 | C1CCC(C1)S | garlic |
| 9 | CCCCCC(=O)OCC | sweet |
| 10 | CC(=O)OC1=C(C=C(C=C1)C=O)OC | sweet |
| 11 | CCCC(C)C(=O)OCC | sweet |
| 12 | CC(=O)OCCC1=CCC2CC1C2(C)C | sweet |
| 13 | CC1=CCC(CC1=O)C(=C)C | sweet |

**Supplementary Table 4** The curated DREAM dataset of high concentration.

| DREAM ID | SMILES | Odor descriptor |
|---|---|---|
| 1 | C1=CC=C(C=C1)C=O | sweet |
| 2 | C1CC(=O)OC2=CC=CC=C21 | sweet |
| 3 | COC1=C(C=CC(=C1)C=O)O | sweet |
| 4 | C(C1C(C(C(O1)O)O)O)O | sweet |
| 5 | COC1=CC2=CC=CC=C2C=C1 | sweet |
| 6 | CC(=O)OCC1=CC=C(C=C1)OC | sweet |
| 7 | CCOC(=O)CC(=O)OCC | sweet,fruity |
| 8 | CCCC(=O)OCC | sweet |
| 9 | CCCCCCCC(=O)OCC | sweet |
| 10 | CCCCCC(=O)OC | sweet |
| 11 | CCCCCCCC(=O)OC | sweet |
| 12 | COC(=O)C1=CC=CC=C1N | sweet |
| 13 | CCCCCCOC(=O)C | sweet |
| 14 | COC1=CC2=C(C=C1)C=CC(=O)O2 | sweet |
| 15 | CCCCC(=O)OCC | sweet,fruity |
| 16 | CCCCCOC(=O)CCC | sweet |
| 17 | CCCCCC1CCCC(=O)O1 | sweet |
| 18 | CCOC1=CC=C(C=C1)C=O | sweet |
| 19 | COC1=CC=C(C=C1)C=O | sweet |
| 20 | CCOC(=O)CCC(=O)OCC | sweet |
| 21 | CCCCCC(=O)OCC | sweet,fruity |
| 22 | CCCCCCC1CCCC(=O)O1 | sweet |
| 23 | CC1CCC(=O)C1=O | burnt |
| 24 | CC(=O)OC1=C(C=C(C=C1)C=O)OC | sweet |
| 25 | CCCCC1C(CC(=O)O1)C | sweet |
| 26 | CCCC(C)C(=O)OCC | sweet,fruity |
| 27 | CC(=O)OCCC1=CCC2CC1C2(C)C | sweet |
| 28 | C1CCCCCCCOC(=O)CCCCCC1 | sweet |
| 29 | CC1=CCC(CC1=O)C(=C)C | sweet |
| 30 | CC=CC1=CC=C(C=C1)OC | sweet |
| 31 | CC=CC1=CC2=C(C=C1)OCO2 | sweet |

AUROC of 1.000, 0.880, and 0.600 for descriptor of 'burnt', 'fruity', and 'sweet', respectively (Supplementary Figure 6).

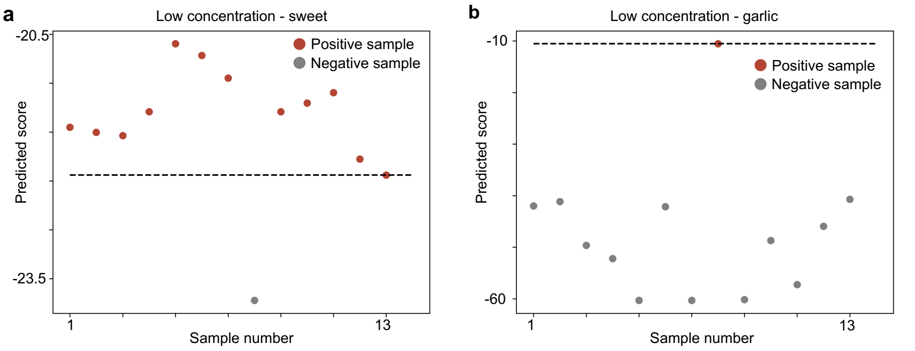


**Supplementary Figure 5** Scatter plot of Mol-PECO's prediction results in a) 'sweet' and b) 'garlic' descriptor for curated DREAM of low concentration.

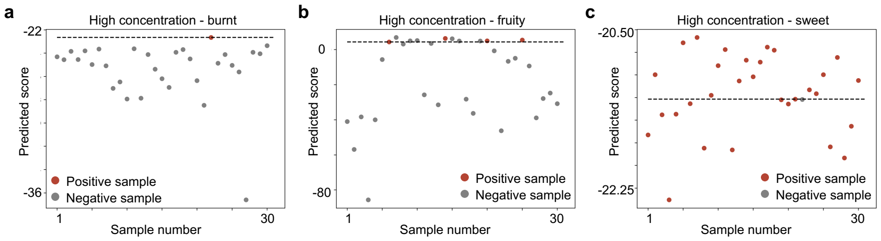


**Supplementary Figure 6** Scatter plot of Mol-PECO's prediction results in a) 'burnt', b) 'fruity', and c) 'sweet' descriptor for curated DREAM of high concentration.

## 2.4 A comparison with previous model

We notice that a graph convolutional model (named RefNet) has been utilized to decode odor percetion with a rather satisfactory performances (reported AUROC / AUPRC of 0.71 / 0.40)[1]. RefNet is constructed similarly with adjacency-GCN (one of our baseline models). RefNet and adjacency-GCN are designed by stacking graph convolutional layers for learning molecular embedding and fully-connected layers for perception prediction, yet the parameters (e.g., the number of hidden neurons and hidden layers) are different. As the codes and the detail of learned model in [1] were not available, we compare Mol-PECO (named Mol-PECO-60Epochs) and RefNet by training them using the same parameter settings disclosed in [1]. Specifically, Mol-PECO-60Epochs and RefNet have been trained by 5-fold cross-validation and each fold has 60 epochs. As the detailed parameters have not been released, we assess the performances of Mol-PECO-60Epochs and RefNet with 9 dropout rates (from 0 to

0.4, step = 0.4 / 9) using the dataset of 5955 molecules (named RefData) released by [1]. Mol-PECO-60Epochs achieves an average AUROC / AUPRC of 0.670 / 0.060, higher than the prediction results of RefNet (AUROC of 0.568 and AUPRC of 0.045, Supplementary Figure 7).

Notably, the baseline model, adjacency-GCN, also shows an inferior performances compared to Mol-PECO using the same parameter settings. These experimental results of GCN-based models seem quite different from the reported performances[1]. We expect that the source of the discrepancy would be the detailed training procedures[1].

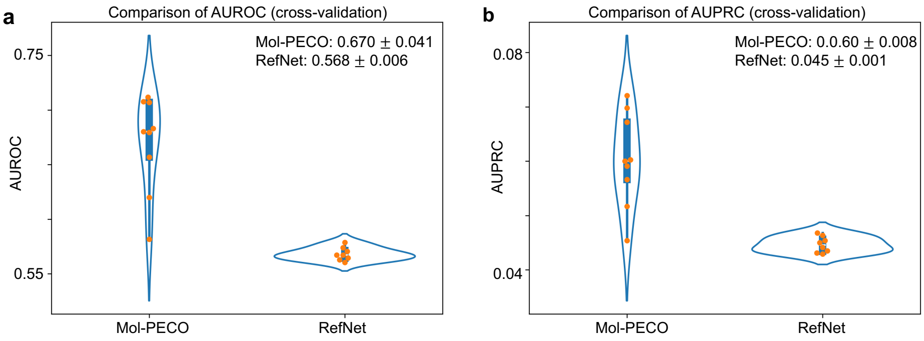


**Supplementary Figure 7** Comparison of results between Mol-PECO-60Epochs and RefNet described in [1].

## 2.5 Dispersion of descriptors in the learned odor space

The distributions of molecules associated with descriptors investigated in Figure 6 are shown as tSNE maps (Supplementary Figure 8). As shown in Supplementary Figure 8a, ‘musk’, ‘amber’, and ‘woody’ have overlapping distributions; the molecules with ‘musk’ are more consolidated within small sub-clusters whereas those with ‘woody’ spread more across the space. Molecules with ‘ethereal’ and ‘fermented’(Supplementary Figure 8b), those with ‘fatty’, ‘waxy’, and ‘oily’(Supplementary Figure 8c), and those with ‘sulfurous’, ‘alliaceous’, ‘garlic’, ‘onion’, ‘meaty’ and ‘roasted’ (Supplementary Figure 8d) are almost colocalized, respectively.

## 2.6 Learning of molecules associated with descriptor ‘musk’

The capability of Mol-PECO to discern molecules associated with the descriptor ‘musk’, despite their varied chemical structures, was systematically examined. Supplementary Figure 9a enumerates the top ten musk-associated molecules, ranked in descending order based on their Mol-PECO prediction scores. The list reveals the tendency of Mol-PECO to favor macrocyclic structures in its identification of musk-like aromas. In stark contrast, Mol-PECO exhibits a notable shortcoming in recognizing nitro musks, evidenced by the listing of the first ten musk-associated molecules, ranked in ascending order of their Mol-PECO prediction scores (Supplementary Figure 9b).

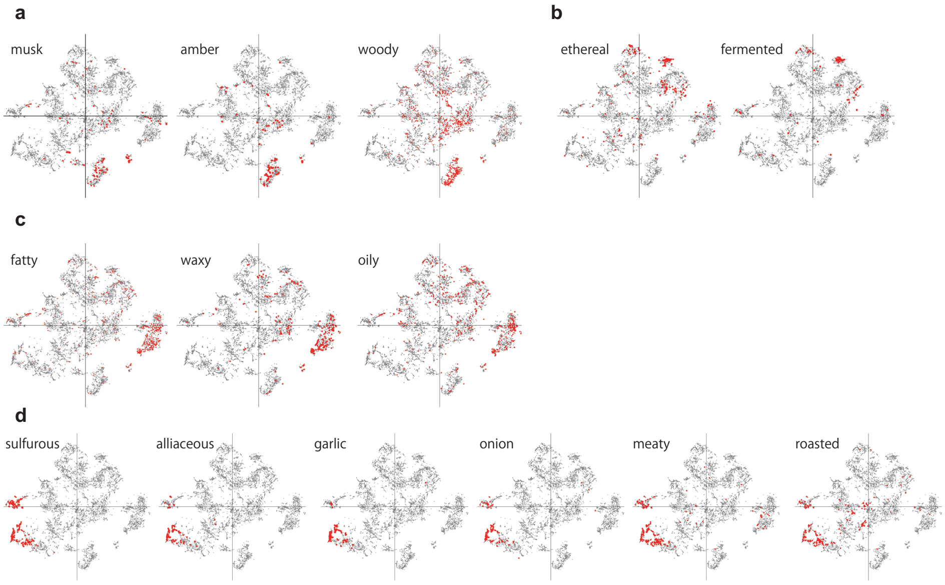


**Supplementary Figure 8** Global view of learned odor space with dimensionality reduction by t-SNE for descriptors forming clusters. (a) The cluster of 'musk', 'amber', and 'woody'. (b) The cluster of 'ethereal' and 'fermented'. (c) The cluster of 'fatty', 'waxy', and 'oily'. (d) The cluster of 'sulfurous', 'alliaceous', 'garlic', 'onion', 'meaty' and 'roasted'.

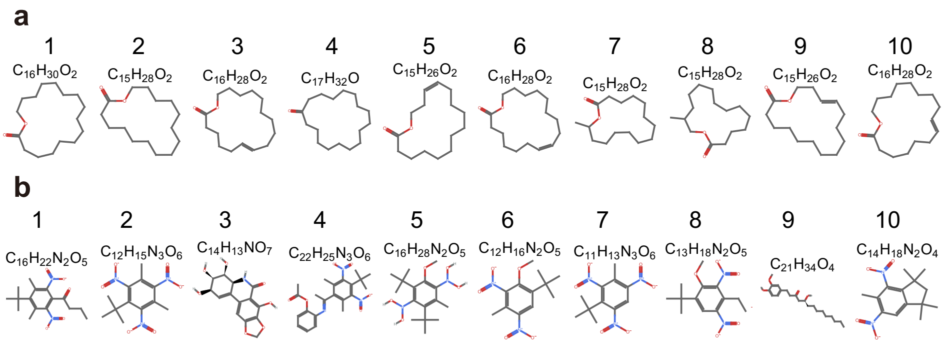


**Supplementary Figure 9** (a) The musk-associated molecules, to which Mol-PECO assign high score of 'musk'. The molecules are sorted in descending order using the prediction score of Mol-PECO. (b) The musk-associated molecules, to which Mol-PECO assign low score of 'musk'. The molecules are sorted in ascending order using the prediction score obtained by Mol-PECO. We omit molecules that are registered as 'musk'-associated molecules in the database but does not produce a proper structure from its SMILES format.

# References

[1] Achebouche, R., Tromelin, A., Audouze, K., Taboureau, O.: Application of artificial intelligence to decode the relationships between smell, olfactory receptors and small molecules. Scientific reports **12**(1), 18817 (2022)
